# Machine learning models for identifying pre-frailty in community dwelling older adults

**DOI:** 10.1186/s12877-022-03475-9

**Published:** 2022-10-12

**Authors:** Shelda Sajeev, Stephanie Champion, Anthony Maeder, Susan Gordon

**Affiliations:** 1 School of Business and Information Systems, Torrens University, 88 Wakefield St, Adelaide, SA 5000 Australia; 2 Centre for Artificial Intelligence Research and Optimisation, Torrens University, Adelaide, Australia; 3 Flinders Digital Health Research Centre, College of Nursing and Health Sciences, Flinders University, Adelaide, Australia; 4 Caring Futures Institute, College of Nursing and Health Sciences, Flinders University, South, 5042 Australia

**Keywords:** Aged, Machine Learning, Elderly, Frailty/diagnosis, Geriatric Assessment, Pre-frailty

## Abstract

**Background:**

There is increasing evidence that pre-frailty manifests as early as middle age. Understanding the factors contributing to an early trajectory from good health to pre-frailty in middle aged and older adults is needed to inform timely preventive primary care interventions to mitigate early decline and future frailty.

**Methods:**

A cohort of 656 independent community dwelling adults, aged 40–75 years, living in South Australia, undertook a comprehensive health assessment as part of the Inspiring Health cross-sectional observational study. Secondary analysis was completed using machine learning models to identify factors common amongst participants identified as not frail or pre-frail using the Clinical Frailty Scale (CFS) and Fried Frailty Phenotype (FFP). A correlation-based feature selection was used to identify factors associated with pre-frailty classification. Four machine learning models were used to derive the prediction models for classification of not frail and pre-frail. The class discrimination capability of the machine learning algorithms was evaluated using area under the receiver operating characteristic curve (AUC), sensitivity, specificity, precision, F1-score and accuracy.

**Results:**

Two stages of feature selection were performed. The first stage included 78 physiologic, anthropometric, environmental, social and lifestyle variables. A follow-up analysis with a narrower set of 63 variables was then conducted with physiologic factors associated with the FFP associated features removed, to uncover indirect indicators connected with pre-frailty. In addition to the expected physiologic measures, a range of anthropometric, environmental, social and lifestyle variables were found to be associated with pre-frailty outcomes for the cohort. With FFP variables removed, machine learning (ML) models found higher BMI and lower muscle mass, poorer grip strength and balance, higher levels of distress, poor quality sleep, shortness of breath and incontinence were associated with being classified as pre-frail. The machine learning models achieved an AUC score up to 0.817 and 0.722 for FFP and CFS respectively for predicting pre-frailty. With feature selection, the performance of ML models improved by up to + 7.4% for FFP and up to + 7.9% for CFS.

**Conclusions:**

The results of this study indicate that machine learning methods are well suited for predicting pre-frailty and indicate a range of factors that may be useful to include in targeted health assessments to identify pre-frailty in middle aged and older adults.

**Supplementary Information:**

The online version contains supplementary material available at 10.1186/s12877-022-03475-9.

## Background

Frailty is a geriatric syndrome associated with impairments to multiple interrelated physiological systems. It results in decreased resilience, increased vulnerability to stressors [1, 2], poorer health outcomes [3] and increased morbidity and mortality [4]. It is preceded by pre-frailty, a transitional period where individuals are at a greater risk of hospitalization, disability and death, but are more likely to return to good health than those who’ve already progressed to a frail state [4].

In Australia, approximately 50% of people aged 65 years and older are classified as pre-frail [5]. As the average lifespan increases and the population ages, the number of Australians at risk of becoming pre-frail or frail also rises, contributing to significant morbidity and mortality, as well as increased health care costs [6]. This trend is occurring globally. A recent systematic review found there was a high risk of frailty and pre-frailty in community dwelling adults across 28 countries [7].

Although frailty is often associated with older age it is not a natural or inevitable consequence of growing older [8]. Frailty results from cumulative exposures across the life course [9], so while the prevalence of frailty increases exponentially across the adult lifespan, the precursors can manifest before middle age [10]. During the pre-frailty period there is an opportunity to intervene to reduce adverse outcomes, but early indicators of a transition into frailty are often detected too late [11, 12].

Known physiological factors contribute to pre-frailty and frailty, but the impact of environmental, social and modifiable lifestyle factors on ageing trajectories is less established [13, 14]. A better understanding of the interrelated risk factors contributing to pre-frailty will provide insight to develop more effective primary health care interventions to delay or prevent adverse decline and improve the health and independence of people as they age [15]. There is a growing interest amongst policymakers and health care services to recognise determinants associated with becoming frail earlier and provide preventative interventions [16]. There is some evidence to show the effectiveness of programs to reverse pre-frailty [4]. Strategies to reliably identify and intervene in early stages of pre-frailty will assist community dwelling adults to preserve their independence for longer [6].

Broadly, tools used for early identification of frailty indicators include objective performance-based measures of physical function, such as the Fried Frailty Phenotype (FFP) [17], subjective clinical classifications, such as the Clinical Frailty Scale (CFS) [18], and self-report measures, such as the Edmonton Frail Scale [19]. Historically these tools were utilized with older people after admission to hospitals or residential facilities, but more recently, they have been validated for identifying pre-frailty indicators in middle-aged, community dwelling adults [20].

Increased screening of environmental, social and lifestyle factors contributing to frailty and pre-frailty amongst middle-aged individuals will assist in developing more accurate modelling of predictors of frailty, more efficient clinical practice guidelines, better targeted health care policy for older people, and improved predictions of health service use patterns. However, further investigation of the validity of pre‐frailty criteria measures is needed to determine the applicability for people younger than 65 years.

This study investigates physiological, anthropomorphic, environmental, social and lifestyle factors that may be associated with pre-frailty outcomes, using machine learning (ML) approaches. ML is a well-known computational technique that offers a means to construct accurate and reliable prediction tools, and to identify the key indicators contributing to the risk of pre-frailty and frailty. ML is a branch of artificial intelligence that is used for data analysis and comprises of numerous algorithms capable of learning from the data, identifying patterns and transforming data into knowledge with minimal or no human intervention [21].

Based on the outcomes of a comprehensive set of health assessments, four of the most popular ML algorithms were used to identify differences amongst individuals classified as not frail and pre-frail using the CFS [18] and FFP [17].

## Methods

### Study design, participants, and features

The Inspiring Health study population comprised of 656 volunteers aged 40–75 years, living independently in one large Australian metropolitan city. The cohort included more women than men (66.8% women) and the average age was 59.9 years (SD 10.6). Details of the participant demographics, study recruitment, inclusion and exclusion criteria, and methodology for data collection has been published previously [15]. Health assessments were performed in accordance with relevant guidelines and regulations.

Participants completed online surveys assessing mental, social and physical health, medical history and health behaviours; and attended an extensive health assessment session to collect objective measures of anthropometry and physiological measures. A complete list of the health assessments collected as part of the Inspiring Health study, and the management of these assessments have been reported [15].

A summary of the cohort characteristics, health measures and assessments used in the analysis is outlined below (Table 1). A detailed description of how these features were defined is also provided in Additional file 1: Appendix A.

**Table 1:** Details of cohort characteristics, health measures and health assessments

**Demographics, environment and social factors**	**Physiological measures**	**Medical history**
•Age	•Audio test	•Current health conditions
•Community participation	•Balance (8 features)	•Distress (2 features)
•Education level	•Blood pressure (2 features)	•Emergency department visits
•Gender	•Cognition test	•History of falls
•Housing type	•Current pain	•Hospitalisations
•Employment status	•Dental health (4 features)	•Medications/supplements
•Income source	•Dexterity	•Near falls
•Living arrangements	•Dizziness	•Recent surgery
•Marital status/partnerships	•Fatigue	•Unintentional weight loss
•Pet ownership	•Foot sensation	**Anthropometry**
•Postcode	•Functional movement	•Body Mass Index (BMI)
•Mode of transport	•screening (6 features)	•Fat mass
**Lifestyle factors**	•Grip strength (3 features)	•Hip circumference
•Alcohol consumption (2 features)	•Reflex test	•Muscle mass
•Current smoking	•Hearing test	•Waist circumference
•Diet quality	•Lung health (2 features)	
•Physical activity level (9 features)	•Pelvic floor health	
	•Sleep quality	
	•Stair climbing	
	•6 Minute Walk Test (6MWT)	

### Outcome measures

The outcome measures used to categorise not frail and pre-frailty was the CFS and FFP. As it was possible to collect objective measures of physical function, self-reported measures of frailty were not collected in this project. Used exclusively by trained health professionals, the CFS categorises individuals across a nine-point scale: Very Fit (1), Well (2), Managing well (3), Vulnerable (4), Mildly frail (5), Moderately Frail (6), Severely Frail (7), Very Severely Frail (8), and Terminally Ill (9) [18]. The first six CFS categories were used for the Inspiring Health assessments: very fit, well, managing well, vulnerable, mildly frail, and moderately frail. Due to small numbers in each category and for simple implementation and analysis, individuals identified as ‘very fit’ and ‘well’ were categorised as not frail, and those considered to be ‘managing well’ and ‘vulnerable’ were categorised as pre-frail. Individuals categorised as ‘mildly frail’ and ‘moderately frail’ were categorised as frail and excluded from the subsequent analysis. This classification of pre-frailty was in part determined by a recent guide from the CFS developers which expands on the definitions of the managing well and vulnerable categories. Managing well includes people with controlled medical problems who are inactive, and the vulnerable category (now renamed to be ‘living with very mild frailty’) includes people who experience symptoms that limit their activities [22]. These categories have been interpreted to equate to an indication of pre-frailty.

The second outcome measure, the FFP, assesses individuals on five criteria [17]:

1. Self-reported unintended weight loss of 4.5 kg or 5% of body mass in the last year.
2. Self-reported exhaustion, reporting most or all of the time in response to the question: “About how often did you feel tired out for no good reason?”
3. Self-reported low levels of physical activity (less than the median recommended amount of time spent walking per week, and no moderate or vigorous intensity physical activity each week)
4. Observed slower than standardised values of Australians gait speed, measured using the individual 6MWT
5. Observed sitting dominant handgrip strength below 10.^th^ percentile normative values (derived from British population studies)

Participants who scored 0 on the FFP scale were categorised as not frail. If they met 1 or 2 criteria; they were categorised as pre-frail. Due to low numbers, individuals who scored 3 or greater were excluded as frail. 

### Missing values, final participant numbers, features and outcome measures

Out of the original 656 participants, 25 individuals were removed from the cohort due to missing outcome data (either FFP or CFS). This led to a sample of 631 (656 – 25) participants. In addition, as the frailty numbers were very small (*n* = 15), individuals categorised as frail according to the FFP (identified as exhibiting 3 or more of the criteria for frailty) were removed that from the analysis. This resulted in 616 (631—15) participants for the analysis. For CFS, the number of individuals in each category were: not frail (*n* = 446), pre-frail (*n* = 170), and for FFP: not frail (*n* = 378), pre-frail (*n* = 238).

The dataset contains 79 input features in addition to the two outcome measures (CFS and FFP). One input variable (employment status) was excluded from the analysis due to greater than 50% missing data. Missing values for the remaining 78 features were imputed using missRanger algorithm which predicts both continuous and categorical missing values using Random Forest algorithm trained on the observed data [23].

### Feature selection

Not all features have significant class discrimination information, and using multivariate high dimensional data is computationally expensive. Feature selection methods can help to remove the irrelevant and redundant features. This could reduce the computational time and improve classification performance. In this study, a popular feature selection method was used: correlation-based feature selection with best fit search method [24]. This feature selection finds the feature subset that shows high correlation with the class label and less correlation with other features. It ignores the irrelevant and redundant features. The selected features were ranked using random forest algorithm according to their contribution to classification.

### Machine learning models

There are three forms of ML: supervised, unsupervised and reinforcement. Supervised models utilise training data with inputs and the desired output labels, while unsupervised models use data without output labels. Reinforcement learning is based on the analysis of past outcomes and feedback. In this study, four widely accepted supervised ML models were used: logistic regression (LR) [25], linear discriminant analysis (LDA) [26], support vector machine with Radial Basis Function (SVM) [27], and random forest (RF) [28]. LR and LDA are two linear classification methods while SVM and RF are more sophisticated ML models that support nonlinear relationships. Most modern deep learning models could not be used for this analysis due to the relatively small size of the dataset.

LR uses a simple classification approach that uses a linear equation with one or more independent features to predict a binary dependent variable. The predicted values are mapped to probabilities using the sigmoid function. LDA finds the orientation vector that maximizes the distance between the two classes after standardizing for within class variance. SVM classifiers determine the optimal separating hyperplane that separates the data into 2 classes with maximum distance margin between both classes. Lastly, RF, a popular ensemble classification method, combines multiple learning algorithms to achieve better performance. RF builds large number of individual decision trees and obtain vote from each tree and classifies the data using majority vote.

### Software

The experimental codes were implemented using Python, RStudio and Weka. Missing data imputation was done in the R 3.6.1 using the missRanger package. Feature selection done in Weka using weka.attributeSelection method, standarisation of features and ML algorithms (LR, LDA, SVM and RF) were implemented using Scikit-learn library python.

### Performance measures

The performance of the ML models was evaluated using area-under-curve (AUC) score, sensitivity, specificity, precision, F1-score and accuracy. The optimal classification threshold was found from the receiver-operating-curve (ROC), which showed the greatest difference in sensitivity and specificity.

### Experimental setting

**Fig. 1:**
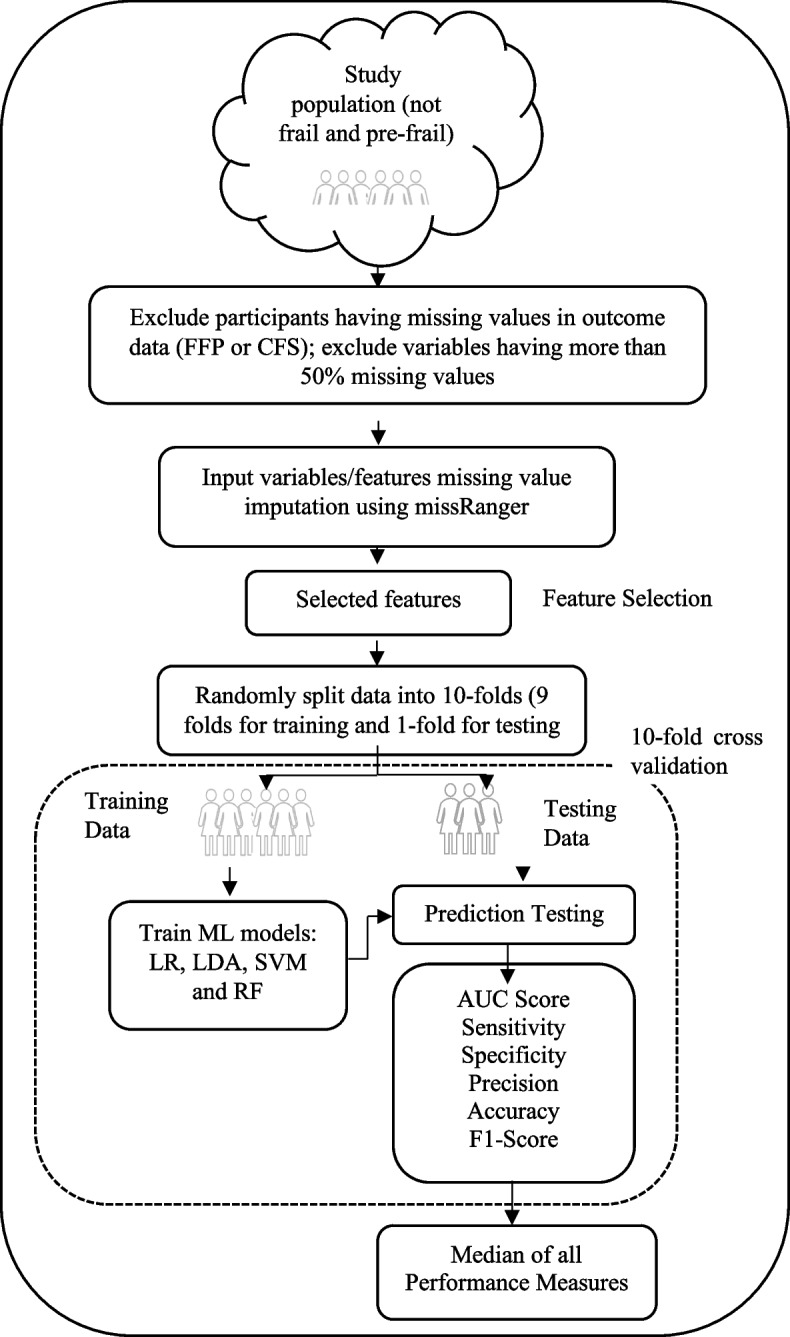
Flowchart describing the overview of the machine learning approach. FFP indicates Fried Frailty Phenotype; Clinical Frailty Scale (CFS); Machine learning (ML); Logistic Regression (LR); Linear Discriminant Analysis (LDA); Support Vector Machine with Radial Basis Function (SVM); Random Forest (RF)

An overview of the ML approach used in this study is reported in Fig. 1. After missing values imputation, the input features values were standardised to ensure each feature had the same influence on the cost function in designing the ML models. Then the data was shuffled and stratified K-fold cross validation approach was used to optimise the models’ robustness and generalisation. The stratified K-fold cross validation approach divides the data randomly into *K* subsets/folds of roughly equal sizes ensuring each fold has the same proportion of outcome class values. Then the model is trained using *K-1* folds and tested with left out *K*th fold. This process is repeated K times, with each fold serving as a testing fold at some point. In this study, we have used tenfold cross validation (K = 10) and the reported values are the median of tenfold cross validation. We have used median as they are robust to outlying predictions.

## Results

### Feature analysis

While analysing 78 features, a combination of 5 features was identified for FFP classification while a combination of 14 features was identified for CFS classification (not frail vs pre-frail).

Lower 6MWT values, poor grip strength, unintentional weight loss, distress, and housing type (for example, living in a house rather than renting a room or living in a low-level care residence) were the selected prominent features for FFP. Having higher body mass index (BMI) scores, lower 6MWT, inactivity, distress, poor balance and functional mobility scores, shortness of breath, lower multiple health conditions, and limited endurance (assessed with stair climbing) were the selected prominent features for CFS. Table 2 shows the most prominent feature subset selected.

**Table 2:** Most prominent feature subset selected using multivariate features analysis based on Weka correlation-based feature selection method with best fit search method for classification of not frail and pre-frail (78 features)

**Fried Frailty Phenotype**		**Clinical Frailty Scale**	
**Features**	**Score**^**a**^	**Features**	**Score**
***Lower 6 Minute Walk Test (6MWT) score (metres walked)***	0.528	Higher Body Mass Index (BMI)	0.179
Weaker grip strength (right hand, sitting)	0.366	***Lower 6MWT score (metres walked)***	0.162
Increased likelihood of unintended weight loss of more than 5 kg	0.058	Fewer hours of vigorous activity (past week)	0.127
***Higher Kessler Psychological Distress Scale (K10) score***	0.025	***Higher K10 score (distress)***	0.091
Housing type (more likely to live in a house)	0.023	Lower functional mobility score (hurdle, left leg first)	0.088
		Higher K9 score (distress)	0.069
		Shortness of breath	0.051
		Diagnosed with any health conditions	0.050
		Lower functional mobility score (raised right leg/left arm)	0.047
		Fewer minutes gardening in the past week	0.038
		Less able to walk up and down 15 stairs without rest	0.032
		Fewer minutes of vigorous activity (past week)	0.031
		Poorer balance assessment (eyes open)	0.021
		Poorer balance assessment (heel toe steps backwards)	0.016

***Bold*** text indicates features found in both the FFP and CFS feature ranking

^a^Score: variable ranking by their contribution to the prediction using Random Forest algorithm. Higher scores indicate greater predictive value

The features relating to assessments included as part of the FFP scale were unsurprisingly prominent in the preliminary investigations. However, the identification of health features not included in the FFP assessments suggested pre-frail adults may exhibit a wider array of physical, global health and social indicators of frailty than those included in the FFP. To identify additional, more subtle indicators of pre-frailty, the performance of four ML models for predicting pre-frailty were retested with a refined dataset of variables. The 15 variables used to assess frailty in the FFP tool were removed from the dataset. Nine of these variables related to levels of activity (hours and minutes spent gardening, in vigorous activity, moderate activity and walking, and total minutes of exercise), three related to grip strength (in right and left hands and combined) and one each measured unintentional weight loss, 6MWT scores and a measure of distress (K9).

While analysing the reduced feature set of 63 features, a combination of 20 features were identified for FFP, and a combination of 19 features were identified for CFS. Table 3 outlines the most prominent feature subset selected from the sub analysis of 63 features. With the FFP related features removed, the features associated with a FFP classification of pre-frailty were much broader. Poor balance, lower levels of functional mobility, impaired cognitive function and lung function, pain, low quality sleep, dental problems, higher BMI and consumption of alcohol were new factors identified as predictors for pre-frailty. Higher K10 scores and housing remained as predictors (Table 3).

**Table 3:** Most prominent feature subset selected using multivariate features analysis based on Weka correlation-based feature selection method with best fit search method for classification of not frail and pre-frail (FFP related features removed, 63 features remain)

**Fried Frailty Phenotype**		**Clinical Frailty Scale**	
**Features**	**Score**^**a**^	**Features**	**Score**
***Higher Body Mass Index (BMI)***	0.157	***Higher BMI***	0.141
Lower Purdue Dexterity Test score (both hands)	0.089	Lower muscle mass	0.116
***Higher Kessler Psychological Distress Scale (K10) score***	0.075	Higher fat mass	0.090
***Higher Pittsburgh Sleep Quality Index (PSQI) Global score***	0.072	***Lower FMS score (raised right leg/left arm)***	0.087
***Lower Functional movement screen (FMS) score (hurdle, left leg first)***	0.059	***Higher K10 score***	0.077
***Lower FMS score (raised right leg/left arm)***	0.056	***Higher PSQI Global score***	0.074
Poorer balance assessment (eyes closed)	0.055	Lower FMS score (hurdle, right leg first)	0.059
***Poorer balance assessment (eyes open)***	0.054	***Poorer balance assessment (eyes open)***	0.054
Lower FMS score (lunge with right leg)	0.053	***Shortness of breath***	0.047
***Bother with pelvic floor problems***	0.050	***Lower FMS score (hurdle, left leg first)***	0.047
***Shortness of breath***	0.047	***Bother with pelvic floor problems***	0.046
Lower FMS score (raised left leg/right arm)	0.044	***Poor lung function test scores***	0.031
***Lower lung function test scores***	0.033	Avoiding/unable to eat some foods because of your mouth, teeth or dentures	0.024
Housing (more likely to live in a house)	0.032	***Lower GPCOG test score***	0.023
Income (more likely to be sourced from a pension)	0.032	Less able to walk up and down 15 stairs without rest	0.021
***Lower General Practitioner assessment of Cognition (GPCOG) test score***	0.024	***Poorer balance assessment (heel toe steps backwards)***	0.019
Self-conscious about their mouth, teeth or dentures	0.021	Mode of transportation (less likely to walk or ride a bike)	0.019
***Poorer balance assessment (heel toe steps backwards)***	0.018	Poorer balance assessment (standing, right leg, eyes open)	0.012
Current pain	0.015	Diagnosed with any health conditions	0.011
Higher consumption of alcohol	0.013		

***Bold*** text indicates features found in both the FFP and CFS feature ranking

^a^Score: variable ranking by their contribution to the prediction using Random forest algorithm. Higher scores indicate greater predictive value

For the CFS, impaired cognitive function and lung function, low quality sleep, mode of transport, higher fat mass and lower muscle mass, and dental problems were new factors identified as predictors for pre-frailty. Multiple health conditions, poor balance, lower levels of functional mobility, higher K10 scores (indicating higher levels of distress), higher BMI, lower endurance (assessed with stair climbing) and housing remained as predictors. Inactivity was no longer associated with being classified as pre-frail as many of the activity variables were removed.

### Prediction accuracy

Table 4 reports the prediction accuracy according to discrimination (AUC score) for each of the four ML models using the selected features and all 63 features for both FFP and CFS. While using all 63 features, random forest achieved the highest AUC scores of 0.701 and 0.776 for FFP and CFS, respectively. All ML models achieved significant improvements in discrimination while using selected features compared to the 63 feature set (from + 2.1% for the random forest model to + 7.4% for support vector machine for FFP and from + 2.4% for random forest to + 7.9% for support vector machine for CFS).

**Table 4:** Ten-fold cross validation: Comparison of the performance of four machine learning models predicting pre-frailty using all 63 features and selected features (subset of 20 features combination for Fried Frailty Phenotype Classification and 19 features combination for Clinical Frailty Scale Classification)

Models	AUC (Selected Features)	AUC (All 63 features)	Difference between selected features and all features
Fried Frailty Phenotype Classification (not frail: pre-frail)			
Logistic Regression	0.704	0.638	+ 6.6%
Linear Discriminant Analysis	0.707	0.637	+ 7.0%
Support Vector Machine	0.700	0.626	+ 7.4%
Random Forest	**0.722**	**0.701**	+ 2.1%
Clinical Frailty Scale Classification (not frail: pre-frail)			
Logistic Regression	**0.817**	0.757	+ 6.0%
Linear Discriminant Analysis	0.805	0.750	+ 5.5%
Support Vector Machine	0.810	0.731	+ 7.9%
Random Forest	0.800	**0.776**	+ 2.4%

### Classification analysis

Using 63 features, the ML models achieved up to 71% accuracy, 82% specificity, 63% sensitivity, 62% precision and an F1-score of 62% for FFP (Table 5). In comparison, the ML models with selected features achieved up to 71% accuracy, 88% specificity, 61% sensitivity, 75% precision, and an F1-score of 61%. The number of subjects correctly classified as not frail (specificity), and the proportion of subjects correctly classified as pre-frail (precision) were significantly better with selected features.

**Table 5:** Ten-fold cross validation: Comparison of prefrailty classification (Accuracy, Sensitivity, Specificity, Precision and F1-Score) using all 63 features and selected features (subset of 20 features combination for Fried Frailty Phenotype Classification and 19 features combination for Clinical Frailty Scale Classification)

Models	Accuracy	Specificity	Sensitivity	Precision	F1-Score
Fried Frailty Phenotype Classification (not frail: prefrail)					
**63 features**					
Logistic Regression	65.6	73.7	51.1	57.1	51.9
Linear Discriminant Analysis	65.1	78.9	53.2	57.9	53.4
Support Vector Machine	65.9	**81.6**	45.8	58.3	52.5
Random Forest	**71.3**	77.4	**62.5**	**61.8**	**61.9**
**Selected features**					
Logistic Regression	69.1	80.3	60.4	67.5	60.0
Linear Discriminant Analysis	69.1	**88.0**	60.4	**75.0**	60.0
Support Vector Machine	69.2	82.3	58.3	66.2	60.5
Random Forest	**70.8**	79.0	**60.5**	65.2	**61.2**
Clinical Frailty Scale Classification (not frail: prefrail)					
**63 features**					
Logistic Regression	**74.0**	75.3	72.6	52.5	55.4
Linear Discriminant Analysis	73.8	76.4	58.8	52.8	56.6
Support Vector Machine	**74.0**	**80.7**	70.6	**53.1**	55.1
Random Forest	72.4	70.8	**73.5**	50.0	**57.7**
**Selected features**					
Logistic Regression	77.9	**81.8**	**79.4**	60.4	61.55
Linear Discriminant Analysis	77.1	77.5	73.5	56.2	62.36
Support Vector Machine	**78.7**	80.0	76.5	**60.9**	**62.38**
Random Forest	74.8	77.8	73.5	53.9	59.94

For CFS, using 63 features, the ML models achieved up to 74% accuracy, 81% specificity, 74% sensitivity, 53% precision and an F1-score of 58%. In comparison, the ML models with selected features had a higher accuracy (79%), specificity (82%), sensitivity (79%), precision (61%) and F1-score (62%).

## Discussion

The results of this study indicate that machine learning methods are well suited for predicting pre-frailty. The ML analysis identified key health assessment measures that contribute to the shift between not frail and pre-frail, as defined using the FFP and CFS. For the final model with 63 features, random forest achieved the highest AUC score of 0.722 for FFP and logistic regression achieved the highest AUC score of 0.817 for CFS. All ML models achieved significant improvements in discrimination while using selected features compared to the 63 feature set. For classification, comparing the 63 feature set to the selected features, for the FFP, the specificity and precision of the selected feature models were significantly better, and for the CFS, selected features models had higher accuracy, specificity, sensitivity, precision and F1-scores.

For feature analysis, when considering all assessment of health (78 features), pre-frailty was most prominently predicted with a range of physical assessments, including 6MWT, grip strength assessments, BMI, mental health, inactivity and functional mobility. Balance, weight loss, shortness of breath and endurance were also identified as factors. For the FFP, the strength of the 6MWT, accounting for 50% of the predictive power, demonstrates the importance of walking speed as a measure of pre-frailty. This finding is supported by the wider literature, walking speed has been shown to be a strong predictor of future health and frailty [29, 30]. Housing was the only nonphysical assessment and that feature accounted for less than 1% of the predictive value. The ML models were able to achieve significantly high predictive accuracy (AUC scores of 0.916 and 0.861 (Additional file 2: Appendix B) for FFP and CFS, respectively), but the findings of these models can only confirm what is already understood about the correlation between physical criteria and frailty. Thus, these preliminary models were superseded by the subsequent analysis with 63 features.

When the 15 FFP assessment features were removed, a broader range of physical factors were associated with pre-frailty, including impaired cognitive function, pain, poor sleep quality, lung function, dental health, pelvic floor distress, higher fat mass and lower muscle mass. As well social indicators associated with pre-frailty were identified, including alcohol consumption, type of housing, source of income and mode of transportation. No single feature accounted for more than 16% of the predictive value.

A function of using the correlation-based feature selection algorithm is that when features with similar correlation with the outcome variable are identified, one variable is dropped from the analysis. In this dataset, the measures of the functional mobility scale were highly correlated, and it is likely that when a variable measuring functional mobility was selected for a model, another functional mobility variable was excluded [24]. This has had no meaningful real impact on the model, but we advise the reader that the left and right leg or arm differences are not likely to be important for interpreting the reported results.

Indicators of balance, stability and strength, and selected anthropomorphic measures (muscle mass and waist, hip circumference) appeared across all models, suggesting greater relative importance of physiological measures in predicting pre-frailty. The K10 scale was the only assessment to be identified across all models to identify a pre-frail classification for the ML analysis. Previous studies have reported similar associations between mental health and pre-frailty in younger people. A Switzerland study found depression to have prognostic value when identifying pre-frailty in people defined as the “youngest old” (aged 65–70 years) [31]. The connection between depression or depressive symptoms in pre-frail individuals has only recently been investigated. Studies report a significant association between depression and pre-frailty and depressive symptoms are associated with an increased risk of becoming pre-frail [32].

The appearance of social and psychological factors associated with pre-frailty suggest the factors contributing to pre-frailty for this cohort are more holistic and complex than age related health decline alone. The relationship between physical frailty and sociodemographic factors has been established, highlighting the importance of collecting social and quality of life measures in investigations of frailty in older adults [33]. Our analysis found age was not an important predictor of classification as not frail and pre-frail. This can be attributed to the nature of the fitted models. Age is likely highly correlated with many of the other variables and, as a result of the feature selection process, was not selected as a feature.

**Fig. 2:**
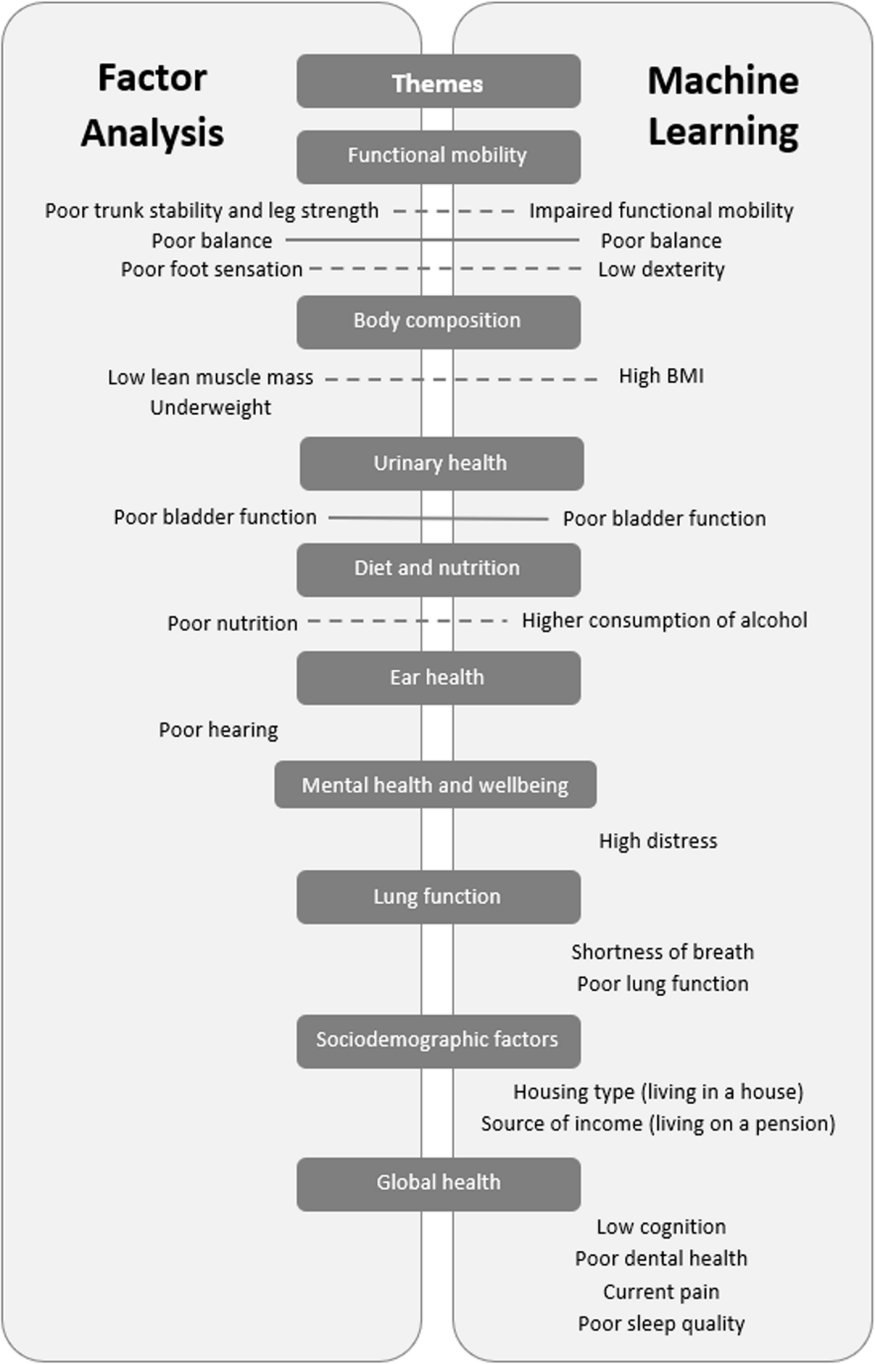
Factor Analysis and Machine learning comparison of features identified to be associated with pre-frailty (defined by Fried Frailty Phenotype), using similar variables

A previous publication reported the results of a factor analysis utilising the Inspiring Health data set. Gordon et al. (2020) investigated factors associated pre-frail and frail independent community-dwelling adults, as determined by the FFP assessments [15]. The outcomes of the factor analysis, based on 25 binary frailty measures, were compared to the outcomes of the ML models derived from 63 variables (Fig. 2). Variables identified to be significantly associated with pre-frailty using a factor analysis approach included poor balance, stability and strength, incontinence, and poor nutrition. In comparison, the ML model identified anthropometric, environmental, social and lifestyle variables in addition to the physiologic ones. This may suggest ML approaches can expose more subtle causal mechanisms that could not been identified with factor analysis.

There is currently no standard screening test for frailty or pre-frailty. Various tools have been developed for a variety of settings and populations [34]. ML enables the development of a more complex modelling strategy, as well as automation and adaptation. It offers an alternative to traditional forms of analysis that allow for a greater number of features to be considered in investigations. ML has increasingly been used in recent years to predict frailty risk in older populations as this approach is ideal for complex, nonlinear clinical problems [35]. Our ML application to detect not frail and pre-frail populations provides greater information about functional decline and the pre-frailty trajectory.

Our ML models were derived from a dataset of 616 individuals. This sample is sufficiently powered for ML modelling approaches, but additional analysis with larger, external data set is required to support our findings and test the validity and generalizability of the models. Due to the small number individuals categorised as frail by the FFP scoring, frail people were removed from the ML analysis. Few frail volunteers are to be expected, given the cohort sample considered of community-dwelling individuals who were fit enough to participate in extensive health assessments. However, the researchers who recruited the Inspiring Health cohort have indicated the sample is generalisable to other urban Australians in terms of age, gender, socioeconomic index, and proportion of people classified as pre-frail [15].

The Inspiring Health study was limited in the number of social determinants that could be reasonably collected. Hence the outcomes may have been biased by the limited psychological testing included in the assessments. A greater range of environmental, social and lifestyle risk indicators should be considered in future Australian ageing cohorts.

## Conclusion

ML has identified sets of indicators that contribute to a classification of not frail or pre-frail. The indicators that performed highly (AUC: 0.722) based on the FFP classification included higher BMI, lower dexterity, greater distress, poorer functional mobility and balance, pelvic floor bother and shortness of breath. Self-reported CFS classification performed highly (AUC: 0.817) in cases characterised by a higher BMI and fat mass, decline in muscle mass and functional mobility, greater distress, poorer balance, shortness of breath and pelvic floor bother. The use of ML has identified different categorisations between not frail and pre-frail participants than previous factor analysis which may suggest ML approaches can expose more subtle casual mechanisms that were not identified with factor analysis.

## Supplementary Information

**Additional file 1: Appendix A.**

**Additional file 2: Appendix B.**

## Data Availability

The datasets analysed during the current study are available from the corresponding author on reasonable request.

## Abbreviations

FFP: Fried Frailty Phenotype
CFS: Clinical Frailty Scale
ML: Machine Learning
6MWT: Six Minute Walk Test
LR: Logistic regression
LDA: Linear discriminant analysis
SVM: Support vector machine
RF: Random forest
AUC: Area-under-curve
ROC: Receiver-operating-curve
FMS: Functional mobility score
BMI: Body mass index
RBF: Radial basis function
PDT: Purdue Dexterity Test
PSQI: Pittsburgh Sleep Quality Index
