# Supplementary material for: Machine learning models for identifying pre-frailty in community dwelling older adults
Source: BMC Geriatr. 2022 Oct 12;22:794. doi: 10.1186/s12877-022-03475-9 (PMC9554971; doi:10.1186/s12877-022-03475-9)
Supplement: Supplementary file 1 — Additional file 1: Appendix A. [file 12877_2022_3475_MOESM1_ESM.docx]

**Table 6:** All variables included in the machine learning models (features in bold were excluded from the 63 feature models)

|  | **Features** | **Measure/Categories** |
| --- | --- | --- |
| **Demographics, environment and social factors** | | |
| 1 | Age | Age in years at time of assessment |
| 2 | Community participation | 0=No; 1=Yes; 3=Sometimes |
| 3 | Education level | 1=<year 10 2=Year 11 3=year 12 4=Cert 5=Trade 6=Dip 7=Bach 8=Mast 9=PHD 10=Prefer not to say |
| 4 | Gender | 1=Male; 2=Female |
| 5 | Housing type | 1=House; 2=Apartment; 3=Unit; 4=Rented room; 5=Low level care; 6=Other |
| 6 | Income source | 1=Pension; 2=Wage; 3=Salary; 4=Self-employed; 5=Self-funded retiree; 6=Other |
| 7 | Living arrangements | 1=Alone; 2=Partner/spouse only; 3=Partner/spouse/children; 4=Children only; 5=Parents; 6=Ext. family; 7=friends; 8=Lodgers |
| 8 | Marital status/partnerships | 1=Single; 2=Married; 3=De facto; 4=Divorced; 5=Separated; 7=Widowed |
| 9 | Pet ownership | 0=No; 1=Yes; 2=Sometimes; 3=Prefer not to say |
| 10 | Postcode | 5XXX |
| 11 | Mode of transport | 1=Walk; 2=Bicycle; 3=Car; 4=Public; 5=Taxi; 6=Taxi vouchers |
| **Lifestyle factors** | | |
| 12 | Alcohol consumption | 0=No; 1=Yes; 999=Not reported (missing) |
| 13 | Current smoking | 0=No; 1=Yes; 999=Not reported (missing) |
| 14 | Diet quality score | Score out of 9 |
| 15 | **Gardening activity (hours)** | Number of hours |
| 16 | **Gardening activity (mins)** | Number of minutes |
| 17 | **Moderate activity (hours)** | Number of hours |
| 18 | **Moderate activity (mins)** | Number of minutes |
| 19 | Number of standard drinks per week | Number of drinks |
| 20 | **Total exercise (minutes)** | Number of active minutes |
| 21 | **Vigorous activity (hours)** | Number of hours |
| 22 | **Vigorous activity (minutes)** | Number of minutes |
| 23 | **Walking (hour)** | Number of hours |
| 24 | **Walking (minutes)** | Number of minutes |
| **Physiological measures** | | |
| 25 | Audio hearing test | 1=Pass; 0=Fail (Participant passes the test if they pass 7 or more of the 8 frequencies) |
| 26 | Balance (left leg, eyes open) | Number of seconds (max of 5 seconds) |
| 27 | Balance (right leg, eyes open) | Number of seconds (max of 5 seconds) |
| 28 | Balance (heel toe, step backward) | 0=No; 1=Yes; 999=Not reported (missing) |
| 29 | Balance (left leg, eyes closed) | Number of seconds (max of 5 seconds) |
| 30 | Balance (right leg, eyes closed) | Number of seconds (max of 5 seconds) |
| 31 | Balance score (left and right leg, eyes open) | Scored 0 to 10 |
| 32 | Balance score (left and right leg, eyes closed) | Scored 0 to 10 |
| 33 | Cognition test | 8/9=Pass; <8=Fail |
| 34 | Current pain | 0=No; 1=Yes; 999=Not reported (missing) |
| 35 | Dental health status | 1=I have my own teeth; 2=I have false teeth/dentures; 3=I have my own teeth AND false teeth/dentures |
| 36 | Dental health: unable to eat some foods | 1=Very often; 2=Fairly often; 3=Occasionally; 4=Hardly ever; 5=Never |
| 36 | Dental health: self-conscious about teeth | 1=Very often; 2=Fairly often; 3=Occasionally; 4=Hardly ever; 5=Never |
| 38 | Dental health: teeth grinding | 1=Very often; 2=Fairly often; 3=Occasionally; 4=Hardly ever; 5=Never |
| 39 | Dexterity test (Purdue Dexterity Test) | Calculated field - mean of 3 measurements (taken from right, left and both hands) |
| 40 | Diastolic Blood Pressure | mmHg |
| 41 | Dizziness | 0=No; 1=Yes; 999=Not reported (missing) |
| 42 | Fatigue (Borg Exertion scale) | Scored out of 20 |
| 43 | Foot sensation (Left and right) | Scored 0 to 20 |
| 44 | Functional Movement Screening - hurdle step (Left foot over) | 0=No; 1=Yes; 999=Not reported (missing) |
| 45 | Functional Movement Screening - hurdle step (Right foot over) | 0=No; 1=Yes; 999=Not reported (missing) |
| 46 | Functional Movement Screening - rotary stability (lift left leg and right arm) | 0=Pain anywhere in the body; 1=Unable to complete the movement pattern or is unable to even get into the position; 2=Able to complete the movement but must compensate in some way; 3=Performs the movement correctly without any compensation |
| 47 | Functional Movement Screening - rotary stability (lift right leg and left arm) | 0=Pain anywhere in the body; 1=Unable to complete the movement pattern or is unable to even get into the position; 2=Able to complete the movement but must compensate in some way; 3=Performs the movement correctly without any compensation |
| 48 | Functional Movement Screening - lunge on the Mat (Left foot forward) | 0=No; 1=Yes; 999=Not reported (missing) |
| 49 | Functional Movement Screening - lunge on the Mat (Right foot forward) | 0=No; 1=Yes; 999=Not reported (missing) |
| 50 | **Grip strength (left)** | Number of kilograms |
| 51 | **Grip strength (right)** | Number of kilograms |
| 52 | **Grip strength (standardised by age and gender)** | 0=Lower than average; 1=average or higher; 999=Not reported (missing) |
| 53 | Reflex test (ruler drop test) | Calculated field, 0 to 30cm (mean of 3 measurements) |
| 54 | Hearing (need to concentrate when listening) | Score out of 10 (1=Not at all to 10=Strongly agree) |
| 55 | Lung function | 1=Findings within normal ranges; 2=Findings show mild airflow limitation; 3=Findings show moderate airflow limitation. Follow up with GP is recommended; 4=Findings show moderate-severe airflow limitation. Follow up with GP is recommended; 5=Findings show severe airflow limitation. Follow up with GP is recommended; 6=Findings show a restrictive pattern. Follow up with your GP is recommended; 7=Findings show a mixed airflow limitation. Follow up with your GP is recommended; 8=Repeatability criteria not met – interpret results with caution; 9=Test performance difficulties – interpret results with caution |
| 56 | Pelvic floor dysfunction score | Scored 0 to 9 |
| 57 | Pelvic floor problems – amount of bother | Scored 0 to 25 |
| 58 | Shortness of breath (Borg Dyspnoea Scale) | Scored out of 10 |
| 59 | Sleep quality (PSQI score) | Scored out of 21 |
| 60 | Stair climbing (walking up 15 stairs) | 1=Yes; 2=No; 3=I can't manage a staircase anymore, but I can walk outside for 5 minutes without resting; 4=I am in a wheelchair. I can move my own wheelchair for 5 minutes without resting; 5=I am in a wheelchair. I cannot move my own wheelchair for 5 minutes without resting |
| 61 | Strength and balance score (Legs and trunk) | Scored out of 14 |
| 62 | Systolic Blood Pressure | mmHg |
| 63 | **6 Minute Walk Test (6MWT)** | Number of metres |
| **Medical history** | | |
| 64 | Current health conditions | 0=No; 1=Yes; 999=Not reported (missing) |
| 65 | **Kessler Psychological Distress Scale (K9, single item)** | Scored out of 5 |
| 66 | Kessler Psychological Distress Scale (K10 total score) | Scored out of 50 |
| 67 | Emergency department visit(s) in the last year | 0=No; 1=Yes; 999=Not reported (missing) |
| 68 | History of falls (last 6 months) | 0=No; 1=Yes; 999=Not reported (missing) |
| 69 | Hospitalision(s) in the last year | 0=No; 1=Yes; 999=Not reported (missing) |
| 70 | Medications or health supplements | 0=No; 1=Yes; 999=Not reported (missing) |
| 71 | History of near falls in the last year | 0=No; 1=Yes; 999=Not reported (missing) |
| 72 | Recent surgery in the last year | 0=No; 1=Yes; 999=Not reported (missing) |
| 73 | **Unintentional weight loss of 4 kg or more (last 6 months)** | 0=No; 1=Yes; 999=Not reported (missing) |
| **Anthropometry** | | |
| 74 | Body mass index (BMI) | kg/m2 |
| 75 | Fat mass percentage | Percentage fat mass (mean of three measurements) |
| 76 | Hip circumference | Number of centimetres (mean of three measurements) |
| 77 | Muscle mass | Percentage muscle mass (mean of three measurements) |
| 78 | Waist circumference | Number of centimetres (mean of three measurements) |
