# Supplementary material for: Machine learning models for identifying pre-frailty in community dwelling older adults
Source: BMC Geriatr. 2022 Oct 12;22:794. doi: 10.1186/s12877-022-03475-9 (PMC9554971; doi:10.1186/s12877-022-03475-9)
Supplement: Supplementary file 2 — Additional file 2: Appendix B. [file 12877_2022_3475_MOESM2_ESM.docx]

**Table 7:** Ten-fold cross validation: Comparison of the performance of four machine learning models predicting pre-frailty using 78 features and selected features using correlation- based feature selection.

| **Models** | **AUC (Selected Features)** | **AUC**  **(78 features)** | **Difference between selected features and all features** |
| --- | --- | --- | --- |
| Fried Frailty Phenotype Classification (not frail: prefrail) | | | |
| Logistic Regression | 0.884 | 0.868 | +1.6% |
| Linear Discriminant Analysis | 0.883 | 0.853 | +3.0% |
| Support Vector Machine | 0.886 | 0.877 | +0.9% |
| Random Forest | 0.916 | 0.891 | +2.5% |
| Clinical Frailty Scale Classification (not frail: prefrail) | | | |
| Logistic Regression | 0.861 | 0.777 | +8.4% |
| Linear Discriminant Analysis | 0.835 | 0.783 | +5.2% |
| Support Vector | 0.838 | 0.789 | +4.9% |
| Random Forest | 0.823 | 0.786 | +3.7% |

**Table 8:** Ten-fold cross validation: Comparison of classification (Accuracy, Sensitivity, Specificity, Precision and F1-Score) and four machine learning models performance using 78 features and selected features (5 for Fried Frailty Phenotype and 14 for Clinical Frailty Scale) using correlation-based feature selection for predicting pre-frailty using an Australian older adult cohort.

| **Models** | **Accuracy** | **Specificity** | **Sensitivity** | **Precision** | **F1-Score** |
| --- | --- | --- | --- | --- | --- |
| Fried Frailty Phenotype Classification (not frail: prefrail) | | | | | |
| **78 features** | | | | | |
| Logistic Regression | 81.3 | 86.7 | 76.5 | 78.0 | 76.3 |
| Linear Discriminant Analysis | 78.9 | 84.2 | 70.8 | 75.5 | 73.2 |
| Support Vector Machine | 82.0 | 86.7 | 72.9 | 77.8 | 76.3 |
| Random Forest | 87.8 | 93.4 | 81.3 | 88.3 | 82.4 |
| **Selected features** | | | | | |
| Logistic Regression | 86.2 | 94.6 | 75.0 | 89.1 | 80.9 |
| Linear Discriminant Analysis | 85.5 | 92.1 | 75.0 | 85.7 | 80.9 |
| Support Vector Machine | 85.5 | 94.7 | 75.0 | 88.9 | 81.2 |
| Random Forest | 86.2 | 95.6 | 76.6 | 91.3 | 81.2 |
| Clinical Frailty Scale Classification (not frail: prefrail) | | | | | |
| **78 features** | | | | | |
| Logistic Regression | 72.4 | 75.6 | 79.4 | 49.0 | 58.7 |
| Linear Discriminant Analysis | 74.8 | 75.3 | 72.9 | 51.1 | 60.3 |
| Support Vector Machine | 75.6 | 73.3 | 64.7 | 54.1 | 61.8 |
| Random Forest | 76.2 | 78.9 | 75.6 | 56.0 | 58.89 |
| **Selected features** | | | | | |
| Logistic Regression | 77.1 | 79.8 | 76.5 | 55.78 | 64.1 |
| Linear Discriminant Analysis | 77.9 | 78.6 | 75.7 | 56.8 | 63.1 |
| Support Vector Machine | 76.6 | 77.5 | 79.4 | 57.7 | 65.0 |
| Random Forest | 78.2 | 83.3 | 67.7 | 62.4 | 61.1 |
